# Editorial: High Temperature Solid Oxide Cells

**DOI:** 10.3389/fchem.2021.719826

**Published:** 2021-09-21

**Authors:** Junwei Wu, Jae-ha Myung, Dong Ding, Tenglong Zhu

**Affiliations:** ^1^ Harbin Institute of Technology, Shenzhen, China; ^2^ Department of Materials Science and Engineering, Incheon National University, Incheon, South Korea; ^3^ Idaho National Laboratory (DOE), Idaho Falls, ID, United States; ^4^ Nanjing University of Science and Technology, Nanjing, China

**Keywords:** solid oxide cells, solid oxide fuel cells, Perovskite, modeling, cathode

In recent decades, the extensive use of fossil fuels has led to global warming, increasing pressure on environmental protection. Solid oxide cells (SOCs) are promising electrochemical energy conversion and storage devices used at high temperatures (600–1,000°C). SOCs can be operated in fuel cell mode (solid oxide fuel cells or SOFCs mode), where they produce electricity from hydrogen or other energy resources such as hydrocarbons, CO, etc., or they can be operated in electrolysis mode (solid oxide electrolysis cells or SOECs mode), where they produce hydrogen or syngas, etc., from H_2_O and CO_2_ when the electricity is supplied. When operated in both SOFC and SOEC modes reversibly, these can be termed as reversible solid oxide cells or RSOCs.

Fundamentally, two types of SOCs have been developed, the tubular and the planer design. The tubular-type SOFC has long-term stability, whereas the planar-type SOFC has high power density compared to the tubular-type SOFC, which shows favorable properties such as high volumetric power densities and low electrical resistance. Xi et al. estimated various physical parameters inside the planar-type SOFC. The model was constructed in detail to include gas flow, heat transfer, mass transfer, and an electrochemical reaction. Therefore, planar-type SOFCs’ performance is affected by structural parameters (Xi et al.).

In addition, the operating temperature of SOFCs plays a critical role in aspects of catalytic activity, stability, electrical efficiency, fuel flexibility, and durability of the materials. It operates at high temperatures (500–900°C), which has the advantage that it can operate with a wide range of fuels, including hydrogen, methane, syngas, ethanol, biogas, and so on. It is possible to maximize the efficiency over 80% through the combination of heat and power generation (CHP). Xi et al. developed a biomass gasification (BG)-SOFC-CHP system with a power generation of 100 kW. The results showed a significant energy-saving effect. The major goals of this work were analyzing the advantages of the CHP system compared to the traditional energy system (Xi et al.).

The operating temperature of SOFCs can affect the physical and chemical process taking place in the cell. These processes are also influenced by the microstructure. Thornton et al. calculated the impedance data for characterizing the microstructure of a SOFC cathode. They found the effective tortuosity of the microstructure of a SOFC cathode by using electrochemical impedance spectroscopy (EIS) data (Thornton et al.). In the aspect of the catalytic activity of the electrode, the high temperature operation favors the use of a non-precious metal catalyst. Xia et al. conducted theoretical calculations and experiments of the hydrogen oxidation process on Ni-CeO_2_ material. The presence of nickel enhanced H_2_ adsorption and lowered the energy barrier that allowed the separated hydrogen to combine with the surface oxygen of CeO_2_ and form H_2_O. The ECR experiments showed that the H_2_ oxidation rate at the surface was increased with the longer deposition time of Ni, corresponding with the theoretical results (Xia et al.). This provides critical insight into the interface reaction between the catalyst and MIEC substrate. However, the advantages of high temperature SOFC are limited due to cost-ineffectiveness and thermal degradation of materials. To solve this problem, Kim et al. studied electrochemical properties of layered perovskite, changing the chemical compositions. They showed the relationship between the electrochemical properties and chemical composition in an SmBa_1-x_Ca_x_Co_2_O_5+δ_ (SBCCO, x = 0.01, 0.03, 0.1, and 0.2) oxide system. The electrical conductivity of SBCCO was about 460 S/m at 500°C which is an excellent value for lower operating temperature (Kim et al.). Kim et al. also studied layered perovskite with A-site deficiency, and they verified that the electrical conductivity was affected by the stoichiometric compositions. They used Sm_1−x_BaCo_2_O_5+δ_ (x = 0, 0.01, 0.02, 0.03, 0.04, 0.05, 0.10, and 0.15) oxide systems as the cathode materials of SOFC. Sm_0.90_BaCo_2_O_5+δ_ (SBCO_0.90) displayed the lowest area-specific resistance (ASR) values, and metal-insulator transition (MIT) electrical conductivity occurred in all samples (Kim et al.). Finally, the authors also considered a crystal structure able to increase electrical conductivity.

Recently, to improve the catalytic activity at an intermediate operating temperature, the *in-situ* exsolution technique has been proposed. The exsolution method was used to grow a nano catalyst from the perovskite system, and the exsolved nanoparticles were socketed onto the perovskite surface. Sun et al. prepared an Ni-Co alloy decorated with lanthanum chromite oxide (LSC-NiCo) *via* an *in-situ* exsolution process and evaluated its electrochemical performance. They introduced A-site deficiency to maximize the exsolution of the B-site. The electrolyte-supported cell decorated with the Ni-Co nano-alloy showed a maximum power density of 320 mW/cm^2^ in H_2_ at 850°C (Sun et al.). The above performance was higher than a single Ni-doped electrode because Co facilitates the exsolution process in LSC-NiCo. This result indicates the versatile functionality of the materials by showing carbon deposition resistance in a syngas atmosphere.

We collected a number of articles and summarized their subjects as follows: various physical parameters, energy efficiency, impedance, hydrogen oxidation reaction of an anode, compositional change for high conductivity materials, and *in-situ* exsolution technique. This research topic shows the valuable contributions of research in the field of high-temperature SOFCs and provides a new insight for the future developments of a high-performance SOFC system.

**FIGURE 1:**
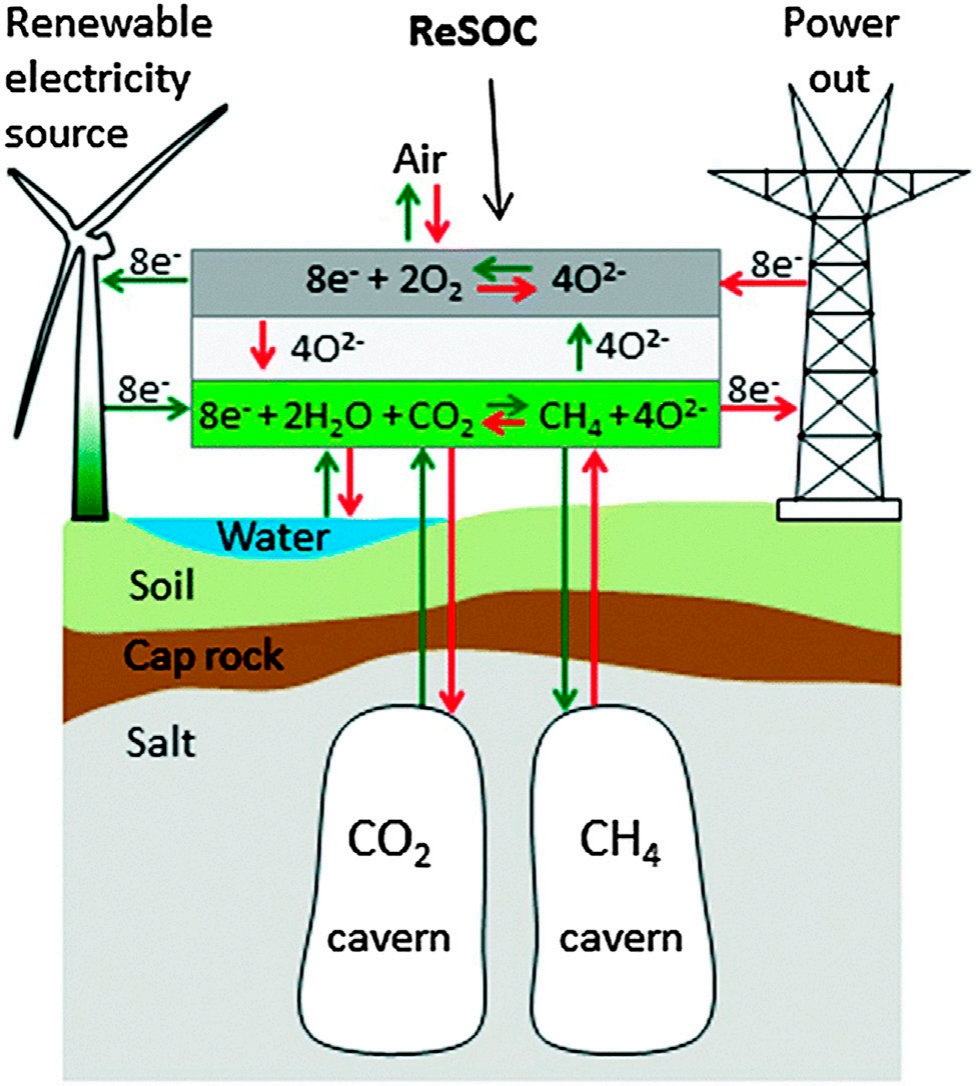
Schematic diagram of the proposed large-scale electricity conversion and storage system.

In the future, renewable energy will need a huge capacity of energy conversion and storage technology because most of the renewable energy will be available as electricity from the most abundant, but intermittent, renewable energy sources, namely electricity from photovoltaic solar cells and wind turbines. By storing the produced gas in existing natural gas grids, the system can create a strong and efficient link between the electricity and gas markets (Jensen et al., 2015; Butera et al., 2019), as shown in Figure 1. In addition, the system is able to operate reversibly using gas from the grid to satisfy the electric power demand. Therefore, more and more research and development will focus on SOECs and RSOCs, including but not limited to, novel materials, design and modeling, operation modes, and so on, which are critical to realize the wide applications of SOC systems.
